# Efficient Acquisition of Fully Human Antibody Genes against Self-Proteins by Sorting Single B Cells Stimulated with Vaccines Based on Nitrated T Helper Cell Epitopes

**DOI:** 10.1155/2019/7914326

**Published:** 2019-12-30

**Authors:** Liangliang Jiang, Tao Jiang, Jianhua Luo, Yanliang Kang, Yue Tong, Xiaoda Song, Xiangdong Gao, Wenbing Yao, Hong Tian

**Affiliations:** Jiangsu Key Laboratory of Druggability of Biopharmaceuticals, State Key Laboratory of Natural Medicines, School of Life Science and Technology, China Pharmaceutical University, Nanjing 210009, China

## Abstract

Single B cell antibody technology is a method for isolating antigen-specific B cells from human peripheral blood and obtaining antibody genes in developing antibody drugs. However, owing to immune tolerance to autoantigen, human autoantigen-specific B cells are difficult to acquire by conventional single B cell technology. In this study, we constructed a nitrated T-cell epitope named NitraTh by incorporating *p*-nitrophenylalanine into a universal T helper epitope. NitraTh had enhanced ability to activate CD4^+^ T cells and can be recognized by CD4^+^ T cells with different HLA class II haplotypes. This NitraTh can also break immune tolerance to autoantigens, such as human epidermal growth factor receptor 2 (HER2) and cannabinoid receptor 1, and induce strong specific IgM^+^ B cell responses *in vitro*. HER2-NitraTh vaccine can also stimulate the generation of HER2-specific IgG^+^ B cells in human immune system mice, which was established by cotransplanting lymphocytes and autologous dendritic cells in immunodeficient mice. We obtained 30 fully human IgG antibody genes by sorting single B cells from the human immune system mice immunized with HER2-NitraTh vaccine. The analysis of antibody genes showed that sorted B cells underwent the extensive somatic mutation of the antibody genes. We randomly selected eight genes for cloning, six of which expressed antibodies that can bind to HER2. Hence, we provided a convenient and effective method in acquiring fully human antibody genes against self-proteins, which can be used in developing therapeutic antibody drugs.

## 1. Introduction

Single B cell antibody technology is a popular approach in developing antibody drugs by sorting antigen-specific B cells from human peripheral blood and obtaining antibody genes. Compared with traditional antibody preparation technologies, this technology is efficient in obtaining monoclonal antibodies (mAbs) with fully human characteristics and rich genetic diversity and maintains a natural pairing of heavy- (VH) and light-chain (VL) variable regions [1]. Cox et al. [2] enriched dengue-enveloped specific B cells from dengue seropositive donors by direct flow cytometry assay (FACS), with a biotinylated dengue-envelope protein, and successfully isolated eight dengue-enveloped specific neutralizing antibodies. Wrammert et al. [3] used the immunoglobulin variable regions that isolated from influenza-specific IgG^+^ B cells to produce >50 human mAbs against influenza virus with high affinity.

However, antigen-specific B cells can only be sorted from the peripheral blood of infected patients or vaccinated volunteers. Hence, the mAbs produced by single B cell antibody technology are limited to target infectious agents, such as *hepatitis B virus*, *West Nile virus*, and HIV [4–6]. No report on the generation of fully human mAbs in noninfectious diseases by this technology has been recorded thus far. Meanwhile, as of 2018, 64 out of 69 mAbs approved by FDA and EMA are used to treat noninfectious diseases in clinical settings, of which oncology and hematology are the most prevalent disease categories [7, 8]. The single B cell technology is limited by the difficulty of obtaining single B cells against autoantigens, which are the common targets in the treatment of tumors or blood diseases, such as human epidermal growth factor receptor 2 (HER2) targeted by trastuzumab and tumor necrosis factor alpha (TNF-*α*) targeted by infliximab.

Breaking self-tolerance to autoantigens is the key to obtaining human specific B cells. When inducible nitric oxide synthase is overexpressed in mammalian cells, tyrosine nitration occurs in several self-proteins. These nitrated proteins not only participate in many pathological processes but also break immune tolerance, activate humoral immunity, and stimulate the production of autoantigen-specific antibodies [9, 10]. Grünewald et al. [11–13] confirmed that the site-specific incorporation of *p*-nitrophenylalanine into self-proteins TNF-*α*, epidermal growth factor, and retinol-binding protein can break immune tolerance and induce a long-lasting specific IgG antibody response.

In our previous study, we designed the nitrated T helper cell epitope named NitraTh) by the site-specific incorporation of *p*-nitrophenylalanine into a universal T helper epitope. This NitraTh not only significantly enhanced the immunogenicity and antitumor activity of HER2 vaccine in mice but also facilitated the production of anti-HER2 antibodies by human peripheral blood mononuclear cells (PBMCs) [14]. Inspired by these results, we sought to establish a method in obtaining autoantigen-specific B cells by stimulating human PBMCs with a NitraTh-based vaccine.

In this work, we evaluated the ability of a series of NitraTh-based vaccines to activate human CD4^+^ T cells. We further confirmed that NitraTh can help various self-antigens to break immune tolerance and induce specific IgM^+^ B cells production *in vitro*. Meanwhile, we developed a functional human immune system in immunodeficient mice that can support humoral immune responses to various antigens and produce specific IgG^+^ B cells. We successfully isolated autoantigen-specific memory B cells that underwent somatic mutation and class switching and obtained >30 fully human IgG antibody sequences by immunizing human immune system (HIS) mice with NitraTh-based vaccine.

## 2. Materials and Methods

### 2.1. Mice and Cell Lines

Female NOD-Prkdc^em26Cd52^ IL2rg^em26Cd22^/Nju (NCG) mice aged 6–8 weeks were purchased from Nanjing Biomedical Research Institute of Nanjing University. All mice were housed in the Laboratory Animal Center of China Pharmaceutical University under specific pathogen-free conditions. All animal experiment protocols were approved by the Laboratory Animal Care and Use Committee of China Pharmaceutical University and properly performed according to the institutional guidelines for the care and use of laboratory animals.

The human breast cancer cell line SK-BR-3 was purchased from Keygen Biotech Co., Ltd. (Nanjing, China). All cells were cultured in RPMI medium 1640 (Gibco, USA) with 10% fetal bovine serum (Gibco, USA) at 37°C in 5% CO_2_.

### 2.2. Synthetic Peptides

All peptides were synthesized by GL Biochem (Shanghai, China) and purified to >98% by reversed-phase high-performance liquid chromatography.

### 2.3. PBMC Isolation and Transplantation

The PBMCs from healthy volunteers, screened negative for HIV-1/2, HTLVI/II, HCV, and HBsAg were isolated by density-gradient centrifugation using lympholyte-H (Cedarlane Laboratories, Canada). To construct HIS mice, we suspended 2 × 10^7^ PBMCs with 200 *μ*L of RPMI 1640 (Gibco, USA) incomplete medium and transplanted into NCG mice by intraperitoneal (i.p.) and intravenous (i.v.) applications. All studies were approved by the Jiangsu Provincial Department for Health (Nanjing, China). Informed consents were obtained from healthy subjects for the collection of blood apheresis samples. Our investigations were performed in accordance with the principles of Declaration of Helsinki.

### 2.4. Screening of Optimal *p*-Nitrophenylalanine Position in Th Epitope by T-DC Cell Coculture In Vitro

The PBMCs were isolated and seeded on 6-well culture plates (Costar, USA) overnight. After discarding the cell suspension, the adherent monocytes were cultured in RPMI 1640 (Gibco, USA) complete medium containing 800 U/ml recombinant human granulocyte-macrophage colony-stimulating factor and 500 U/ml IL-4 (R&D Systems, USA) for 7 days. Culture medium and cytokines were renewed every 2 days. On day 4, antigenic peptides (B, B-T, B-2T to B-11T) were added to a final concentration of 50 *μ*g/mL. On day 6, DCs were stimulated with 40 ng/mL recombinant human TNF-*α* (R&D Systems, USA). After 24 h of stimulation, mature antigen-loaded DCs were harvested carefully. Human naïve CD4^+^ T cells (purity of >95%, viability of >90%) sorted from the same donor by Human Naïve CD4^+^ T Cell Isolation Kit II (Miltenyi Biotec, Germany) were dyed with CFSE (Thermo Fisher Scientific, USA) and then cocultured with antigen-loaded DCs for 7 days. The fluorescence of CFSE was measured by FACS to calculate the proliferation rates of naïve CD4^+^ T cells.

### 2.5. Detection Immunogenetic of NitraTh-Based Vaccines by In Vitro Immunization System

The DCs were induced as above and stimulated with different NitraTh-based vaccines (HER2, HER2-Th, HER2-NitraTh, CB1, CB1-Th, or CB1-NitraTh). Naïve CD4^+^ T cells sorted from the same donor were cocultured with these DCs in the AIM-V medium (Gibco, USA) containing 0.5 ng/mL IL-12 and 1 ng/mL TGF-*β* (R&D Systems, USA) for 7 days. Culture medium and cytokines were renewed every 3 days. On day 5, human naïve B cells (purity of >95%, viability of >90%) that were sorted by Human Naïve B Cell Isolation Kit II (Miltenyi, Germany) were activated with CpG ODN 2006 (InvivoGen, USA) and NitraTh-based vaccines mentioned above. On day 7, B cells were harvested and cocultured with DC-T cells. At 12 days later, the culture supernatants and cells were collected, and autoantigen-specific antibodies and antibody-secreting B cells were measured by ELISA and ELISpot assay, respectively.

### 2.6. Immunization

A total of 2 × 10^7^ PBMCs were sorted and stimulated with 100 *μ*g keyhole limpet hemocyanin (KLH; Sigma-Aldrich, USA), chicken ovalbumin (OVA; Sigma-Aldrich, USA), HER2-Th, or HER2-NitraTh in RPMI 1640 complete medium overnight. Afterward, the PBMCs were harvested and suspended with 200 *μ*L of RPMI 1640 incomplete medium and then transplanted into NCG mice. On day 7, mice were immunized with antigen-loaded autologous DCs (5 × 10^5^) by i.v. injection or with 50 *μ*g antigens in 100 *μ*L of PBS mixed with an equal volume of alum adjuvant (Thermo Fisher Scientific, USA) by i.p. injection. At 14 days later, mice were boosted with 50 *μ*g antigens in 100 *μ*L of PBS mixed with 100 *μ*L of alum by i.p. injection. At 7 days after the last immunization, the mice were euthanized, and tissue samples were collected.

### 2.7. ELISA Assay

The sera of the immunized mice were collected weekly and used in the analysis of KLH, OVA, and HER2-specific human antibody titers by using ELISA. The 96-well ELISA plates were coated with either KLH, OVA, or HER2 (10 *μ*g/mL) and incubated at 4°C overnight. After washing 5 times with PBST (PBS containing 0.05% Tween 20), the plates were blocked with PBS containing 6% BSA (BioFroxx, Germany) at 37°C for 2 h. The serum was diluted 400 or 800 times with PBS containing 2% BSA. Then, the plates were incubated with diluted serum at 37°C for 2 h. After washing 5 times with PBST, the plates were incubated with HRP-conjugated goat antibodies (1 : 10000 *v*/*v*) against human IgM or IgG (Thermo Fisher Scientific, USA) at 37°C for 1 h. Finally, the TMB substrate chromogenic solution (Solarbio, China) was added and incubated at 37°C for 15 min. The reaction was termination with 1 M sulfuric acid solution. The results were measured at 450 nm with 630 nm reference by a microplate reader.

HER2-specific antibodies or CB1-specific antibodies in culture supernatant of *in vitro* immunization system were detected in the same way.

### 2.8. ELISpot Assay

Splenocytes from the immunized mice were collected on day 28 and used to detect KLH, OVA, or HER2-specific antibody-secreting B cell generation. In brief, multiscreen filter plates (Dakewe Biotech Co., China) were coated with either KLH, OVA, or HER2 (50 *μ*g/mL) and incubated at 4°C overnight. A total of 2 × 10^5^ splenocytes were added to each well and incubated at 37°C and 5% CO_2_ overnight. Then, the number of specific B cells was measured by Human IgG or IgM ELISpot^BASIC^ kit (Mabtech AB, Sweden) according to the manufacturer's instructions.

Human B cells from *in vitro* immunization system or HEK293 cells that transfected with antibody genes were collected and detected by the same way.

### 2.9. Antibodies and Flow Cytometry

Cells (1 × 10^6^) were stained with certain antibodies in 100 *μ*L of PBS containing 0.2% BSA for 30 min on ice protected from light and analyzed by FACS.

The proportion of different human lymphocyte subsets in splenocytes of immunized mice were determined using the following mAbs for surface staining: PerCP-Cy5.5 Mouse Anti-Human CD45 (HI30), PE Mouse Anti-Human CD19 (HIB19), Mouse Anti-Human CD4 (RPA-T4), APC Mouse Anti-Human CD69 (FN50), FITC Mouse Anti-Human CD4 (RPA-T4), PerCP-Cy5.5 Rat Anti-Human CXCR5 (RF8B2), PE Mouse Anti-Human ICOS (DX29), PE Mouse Anti-Human CD11c (B-ly6), and FITC Mouse Anti-Human HLA-DR (G46-6). To sort HRE2-specific and naïve single B cells, we used the following mAbs for surface staining: FITC Mouse Anti-Human CD3 (UCHT1), PE Mouse Anti-Human CD19 (HIB19), and PerCP-Cy™5.5 Mouse Anti-Human CD27 (M-T271). All of the mAbs above were purchased from BD Biosciences (USA).

Single-cell sorting was performed on the HRE2-specific B cells, and APC-labeled peptide probes against HER2 B epitope were prepared. In brief, the biotinylated HER2 B epitope peptides were mixed with streptavidin-labeled APC at 33 : 1 and reacted at 4°C overnight. The reaction system was filtered with centrifugal filter unit (Amicon Ultra-0.5, USA) for the removal of excess biotinylated HER2 B epitope peptides and further concentrated to 15 nM.

HER2 antibody from clone B was tested following mAbs for surface staining: HER2/ErbB2 Rabbit Polyclonal antibody (Proteintech, USA) and Alexa Fluor 488 goat anti-rabbit IgG (H+L) (Beyotime, China).

### 2.10. Immunohistochemistry and Immunofluorescence

At sacrifice, the spleens and lymph nodes were fixed in 4% paraformaldehyde, dehydrated with graded alcohol, and embedded in paraffin. Then, 5 *μ*m tissue sections were cut. After treatment with heated citrate buffer for antigen retrieval and incubated in 0.3% hydrogen peroxide to inhibit the endogenous peroxidase, the sections were stained with mouse anti-human CD3 and CD19 antibodies. Immunohistochemistry was performed using the EnVision+ system peroxidase staining procedure (Dako, CA, USA). At the end of the procedure, the sections were reacted with 3-diaminobenzidine and counterstained with hematoxylin for 5 min. For immunofluorescence assay, then sections were stained with mouse anti-human CD11c and rabbit anti-human CD3 antibodies and then incubated with Alexa Fluor 488- (green) conjugated goat anti-mouse IgG and Cy3- (red) conjugated goat anti-rabbit IgG. Then, the slides were scanned by a digital slide scanner. All the antibodies were purchased from Servicebio (China).

### 2.11. Single Cell Real Time- (RT-) PCR and Immunoglobulin Gene Sequencing

CD3^−^CD19^+^CD27^+^HER2^+^ B cells were sorted from the spleens of mice immunized with HER2-NitraTh. CD3^−^CD19^+^ naïve B cells were sorted from peripheral blood in healthy donors. These cells were sorted directly into 96-well PCR plates. The plates were sealed and immediately frozen on dry ice before sent to Jinweizhi Biotechnology Co., Ltd. High-throughput Sequencing Center (Suzhou, China). The cDNAs of samples were acquired by reverse transcription, and Ig variable region genes were subsequently amplified by nested PCR. The sequences were analyzed by IgBLAST comparison with GenBank to identify frame region and complementarity-determining region (CDR) with highest identity.

### 2.12. Vector Cloning and Recombinant Antibody Production

Naturally paired VH and VL were cloned into expression vectors containing human IL2 signal sequence and human constant regions (IgG1 VH constant region or Ig Kappa Light constant domain). Then, IgH and corresponding IgL chain expression vector DNA were transiently transfected into HEK293 cell lines using Lipofectamine 3000 (Invitrogen, USA). The IgG antibodies from clone B were expressed and purified using Hitrap Protein A HP (GE Healthcare, USA).

### 2.13. Statistical Analysis

Statistical analysis was performed by unpaired Student's *t* test or one-way ANOVA. The differences were considered statistically significant if the *p* value was <0.05. All calculations were performed using the Prism software 5.0 (GraphPad).

## 3. Results

### 3.1. Introduction of *p*-Nitrophenylalanine Allowed Th Epitopes to Activate Human Naïve CD4^+^ T Cells

In the previous study, we introduced *p*-nitrophenylalanine at various sites of a universal T helper cell epitope and fused with B cell epitopes of HER2 (378–394; named B) to form a series of NitraTh-based vaccines named B-2T to B-11T. B-T contained T help epitope without *p*-nitrophenylalanine. We found the immunogenicity of NitraTh-based vaccines in mice was closely related to the introduction site of *p*-nitrophenylalanine and MHC class II molecules [14]. Considering the differences in the structure and function of MHC class II molecules between human and murine, we evaluated the ability of NitraTh-based vaccines to stimulate human CD4^+^ T cell proliferation.

**Figure 1:**
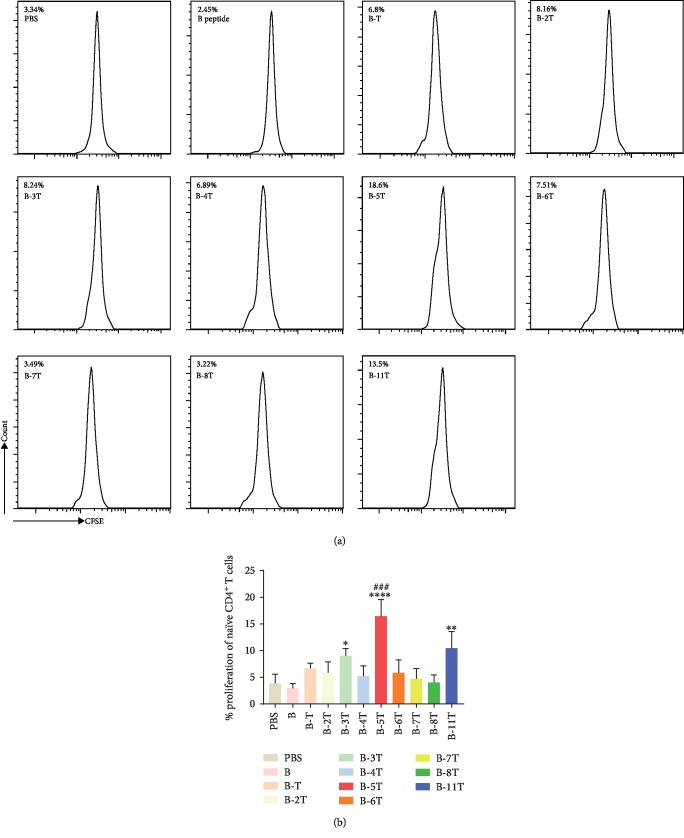
Introduction of *p*-nitrophenylalanine allows Th epitopes to activate human naïve CD4^+^ T cells. (a) CFSE-labeled human naïve CD4^+^ T cells were cocultured with autologous DCs pulsed by various NitraTh-based vaccines for 7 days and analyzed by FACS. One of representative experiments was shown. (b) The results of (a) were analyzed. Data from three independent experiments. ^∗^*p* < 0.05, ^∗∗^*p* < 0.01, and ^∗∗∗∗^*p* < 0.0005 compared with (b); ^###^*p* < 0.001 compared with B-T.

**Figure 2:**
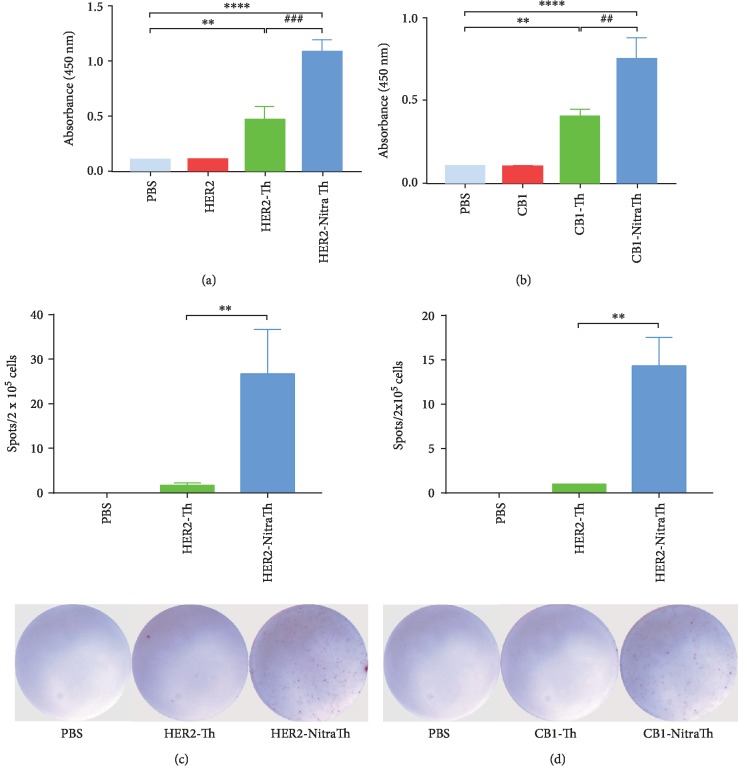
NitraTh contributes to antigen-specific IgM^+^ B cells formation in vitro. (a) Detection of HER2-specific IgM in culture supernatants of DC-T-B cell coculture system by ELISA assay. ^∗∗^*p* < 0.01 and ^∗∗∗∗^*p* < 0.0005 compared with HER2; ^###^*p* < 0.001 compared with HER2-Th. (b) HER2-specific IgM-secreting B cells were detected by ELISpot assay. A total of 26.7 ± 8.2 HER2-specific B cells were present in every 2 × 10^5^ B cell of the coculture system. ^∗∗^*p* < 0.01 compared with HER2-Th. (c) Detection of CB1-specific IgM in culture supernatants of DC-T-B cell coculture system by ELISA assay. ^∗∗^*p* < 0.01 and ^∗∗∗∗^*p* < 0.0005 compared with CB1; ^##^*p* < 0.01 compared with CB1-Th. (d) CBR1-specific IgM-secreting B cells were detected by ELISpot assay. A total of 14.3 ± 2.6 CB1-specific B cells were present in every 2 × 10^5^ B cell of the coculture system. ^∗∗^*p* < 0.01 compared with CB1-Th.

**Figure 3:**
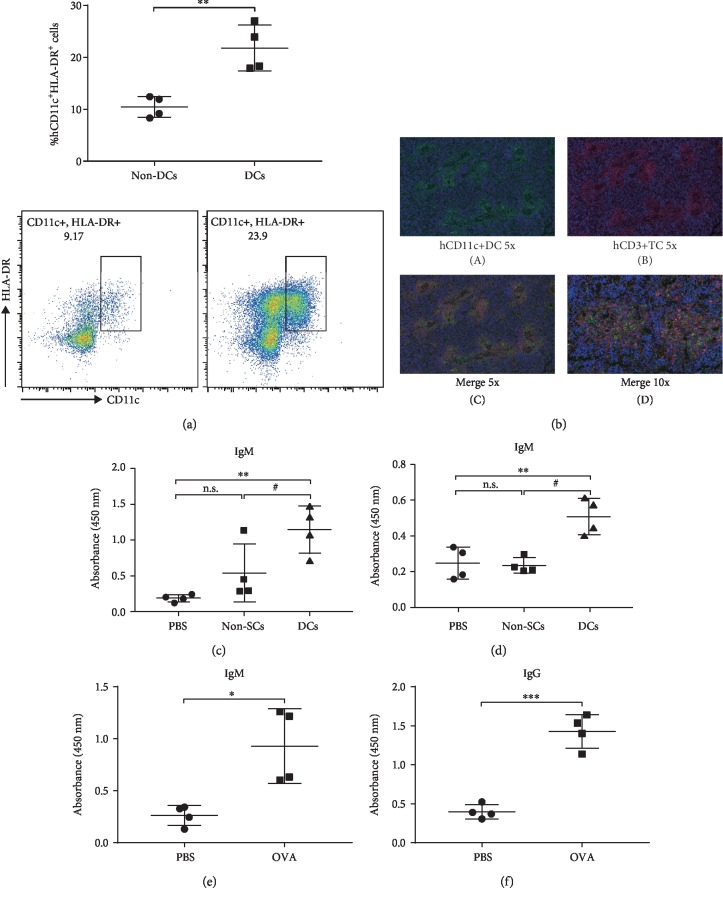
Cotransplantation of autologous DCs allowed HIS mice to produce specific human IgG against various antigens. NCG mice were engrafted with PBMCs on day 0 and then immunized with KLH-loaded DCs on day 7 and KLH-mixed alum adjuvant on day 21 or with KLH-mixed alum adjuvant on days 7 and 21. Data were expressed as mean ± SD (*n* = 4). (a) Splenocytes were harvested from vaccinated mice on day 28, and the percentages of human DCs (gated as hCD11c^+^HLA-DR^+^) were measured by FACS. ^∗∗^*p* < 0.01 compared with non-DCs. (b) (A, B) Immunofluorescent examination of hCD11c^+^ DCs (green) and hCD3^+^ T cells (red) in the spleen sections from HIS mice immunized with KLH-loaded DCs. Original magnification: ×5. (C, D) Immunofluorescent colocalization of autologous hCD11c^+^ DCs and hCD3^+^ T cells. Original magnification: ×5 (C) and ×10 (D). (c, d) The sera of vaccinated mice were collected on day 21, and the production of KLH-specific human antibodies was detected at a 1 : 800 titer by ELISA assay. n.s.: not significant. ^∗∗^*p* < 0.01 compared with PBS; ^#^*p* < 0.05, ^##^*p* < 0.01 compared with non-DCs. (e, f) Sera were collected on the 14th day after immunization with OVA-loaded DCs, and the production of OVA-specific human antibodies was detected by ELISA assay. ^∗^*p* < 0.05, ^∗∗∗^*p* < 0.001 compared with PBS.

**Figure 4:**
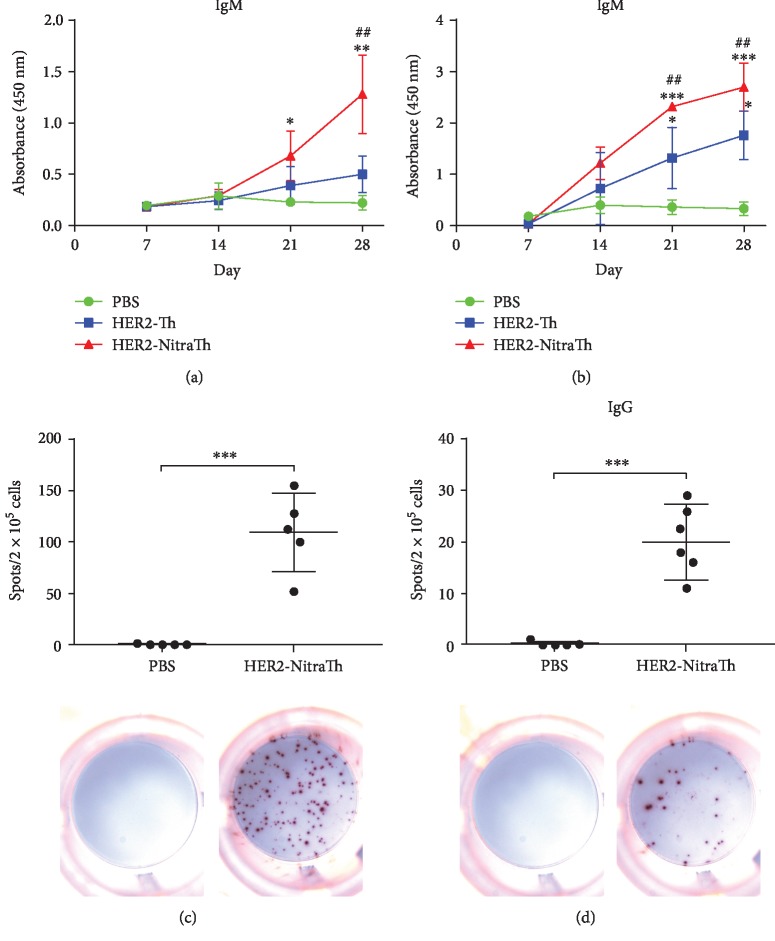
HER2-NitraTh elicits HER2-specific IgG^+^ B cells in vivo. NCG mice were engrafted with PBMCs on day 0 and DCs on day 7 to build DC-HIS mice. Then, DC-HIS mice were immunized with HER2-NitraTh or HER2-Th on days 7 and 21. (a, b) The sera of vaccinated mice were collected weekly, and the production of HER2-specific human IgM and IgG were evaluated at 400 titer by ELISA. ^∗^*p* < 0.05, ^∗∗^*p* < 0.01, and ^∗∗∗^*p* < 0.001 compared with PBS; ^#^*p* < 0.05 and ^##^*p* < 0.01 compared with HER2-Th. (c, d) Splenocytes were harvested from vaccinated mice on day 28, and the generation of HER2-specific IgM-secreting B cells and IgG-secreting B cells was detected by ELISpot assay. ^∗∗∗^*p* < 0.001 compared with PBS. Data were expressed as mean ± SD (*n* = 5).

**Figure 5:**
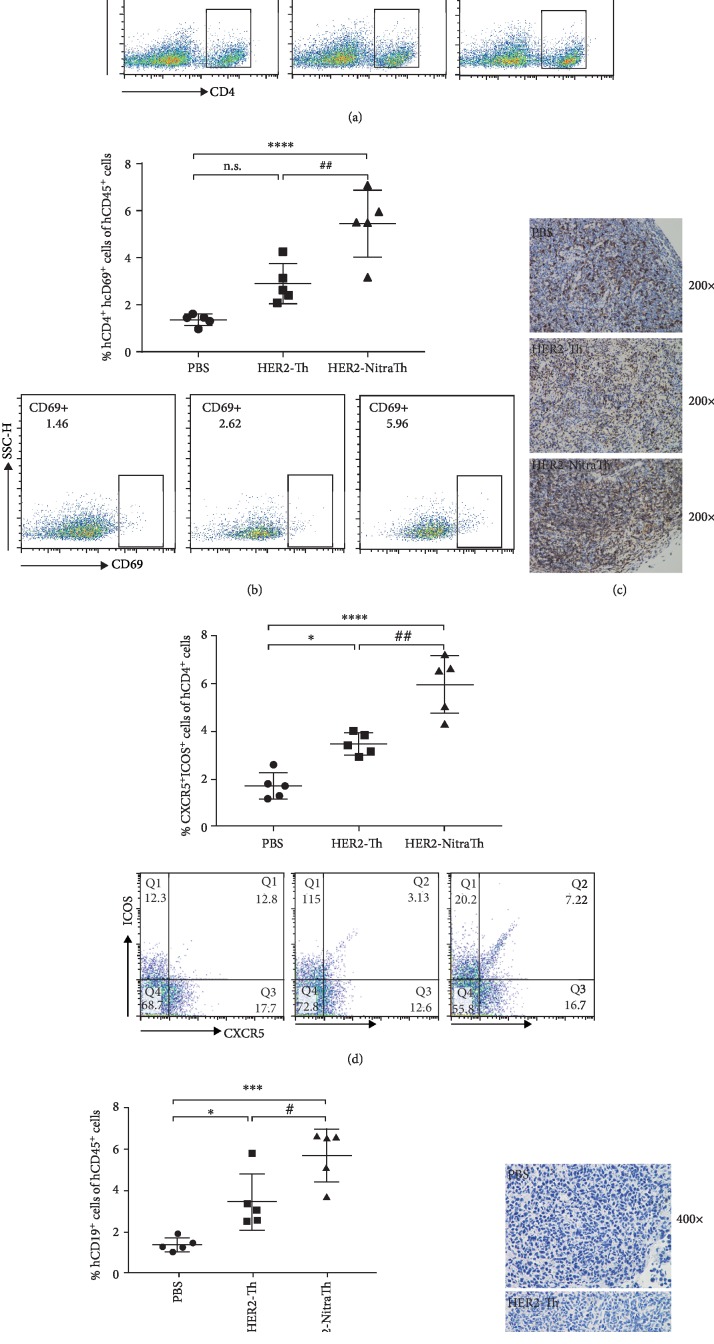
NitraTh improved peripheral human T cell activation and induces differentiation into Tfh cells in vivo. NCG mice were engrafted with PBMCs on day 0 and DCs on day 7 to build DC-HIS mice. Then, DC-HIS mice were immunized with HER2-NitraTh or HER2-Th on days 7 and 21. Splenocytes and lymph nodes were harvested from vaccinated mice on day 28. Data were expressed as mean ± SD (*n* = 5). (a, b, and e) FACS determined the percentages of multilineage human immune cells (pregated on hCD45^+^ cells as the total mononuclear cells) in the spleen. Helper T cells (gated as hCD4^+^), activated helper T cells (gated as hCD4^+^hCD69^+^), and B cells (gated as hCD19^+^). (d) FACS determined the percentages of Tfh cells (gated as CXCR5^+^ICOS^+^, pregated on hCD4^+^ cells) in the spleen. (c, f) Immunohistochemistry analyzed the distribution of hCD3^+^ T cells (brown) and hCD19^+^ B cells (brown) in the lymph node sections. Original magnification: ×400. n.s.: not significant. ^∗^*p* < 0.05, ^∗∗∗^*p* < 0.001, and ^∗∗∗∗^*p* < 0.0005 compared with PBS; ^#^*p* < 0.05 and ^##^*p* < 0.01 compared with HER2-Th.

We stimulated the naïve CD4^+^ T cells sorted from PBMCs with NitraTh-based vaccines for 1 week and detected the proliferative response of naïve CD4^+^ T cells. As shown in Figure 1, B-3T, B-5T, and B-11T can significantly activate naïve CD4^+^ T cells. The proliferation rate of naïve CD4^+^ T cells induced by B-5T was also significantly higher than that induced by B-T (*p* < 0.001), with the rate up to 16.4% ± 1.8%. In subsequent experiments, B, B-T, and B-5T were renamed as HER2, HER2-Th, and HER2-NitraTh, respectively.

Generally, the activation of naïve CD4^+^ T cells by Th epitopes is restricted by HLA class II molecules. To verify that HER2-NitraTh is of high potency universality in human, we sorted naïve CD4^+^ T cells from different volunteers and stimulated with HER2-NitraTh. As shown in Table 1, HER2-NitraTh can activate naïve CD4^+^ T with different HLA class II haplotypes; six out of the seven samples had significant proliferative responses after the stimulation with HER2-NitraTh.

### 3.2. NitraTh Contributed to Antigen-Specific IgM^+^ B Cell Formation In Vitro

To determine whether NitraTh can break immune tolerance to autoantigens, we administrated an *in vitro* immunization approach to detect the occurrence of specific immune responses. ELISA assay confirmed that HER2-NitraTh can remarkably induce the production of HER2-specific IgM antibodies compared with HER2-Th (*p* < 0.001, Figure 2(a)). Consistent with these results, ELISpot assay showed that HER2-specific IgM-secreting B cells were significantly increased when naïve B cells were stimulated with HER2-NitraTh (*p* < 0.01, Figure 2(b)).

To explore whether NitraTh played a similar role in different autoantigens further, we fused the extracellular region of Cannabinoid receptor 1 (CB1) (254-272) with universal Th epitope or NitraTh and named CB1-Th or CB1-NitraTh. As shown in Figure 2(c), CB1-NitraTh induced a significantly higher level of CB1-specific IgM antibodies than CBR1-Th (*p* < 0.01). CB1-NitraTh can also effectively stimulate the production of CB1-specific IgM-secreting B cells (Figure 2(d)). Hence, NitraTh can break immune tolerance to autoantigen and induce the production of autoantigen-specific IgM^+^ B cells.

### 3.3. Cotransplantation of Autologous DCs Allowed HIS Mice to Produce Specific Human IgG against Various Antigens

Although NitraTh can break immunity tolerance, inducing IgG^+^ B cells *in vitro* was difficult due to the limitations of culture conditions. To provide an environment for the differentiation and maturation of antigen-specific IgG^+^ B cells, we transplanted PBMCs into NCG mice to establish a functional human immune system. Considering that DCs, which are directly sorted from PBMCs, are extremely scarce and in an immature state with poor endocytosis and processing capabilities [15, 16], we induced monocytes from PBMCs to differentiate into DCs *in vitro* and then transplanted antigen-loaded DCs into HIS mice to construct DC-HIS mice. At 7 days after PBMC transplantation, the mice were transplanted with KLH-loaded autologous DCs (designated DC group). KLH-mixed alum adjuvant named the non-DC group was administered as a control. On the 21st day after primary immunization, we observed a significant increase in human DCs (hCD11c^+^HLA-DR^+^) in the spleen of the DC group (*p* < 0.01, Figure 3(a)), thereby indicating the successful engraftment and migration of DCs. Immunofluorescent assay showed that donor-matched human DCs and T cells clustered together in certain areas of the spleen (Figure 3(b)), thereby revealing the possible occurrence of DC-T cell interactions and antigen-specific activation of T cells *in vivo*. A significantly high level of KLH-specific human IgM was detected in the serum of DCs group compared with the non-DC group (*p* < 0.05, Figure 3(c)), which agreed with the result above. Only KLH-loaded DCs can induce a considerable KLH-specific human IgG antibody titer in HIS mice among all the groups (Figure 3(d)).

To determine if the cotransplantation of DCs can trigger specific B cell response against multiple antigens further, we immunized mice with chicken OVA-loaded DCs. As shown in Figure 3(e), OVA-specific human IgM can be induced by OVA-loaded DCs. The level of OVA-specific human IgG also showed significant difference (*p* < 0.001) between OVA-loaded DC and control groups (Figure 3(f)). Hence, the HIS mice model on the basis of DC cotransplantation can produce human IgG antibodies against model antigens, thereby providing a reliable approach to acquire human IgG^+^ B cells.

### 3.4. HER2-NitraTh Elicited HER2-Specific IgG^+^ B Cells In Vivo

To obtain autoantigen-specific IgG^+^ B cells further, we immunized DC-HIS mice with Her2-NitraTh or Her2-Th. The HER2-specific antibody titer was measured by ELISA assay weekly. As shown in Figure 4(a), HER2-NitraTh induced a strong IgM^+^ B cell response on day 28, and the HER2-specific IgM antibody titer elicited by HER2-NitraTh was significantly higher than that elicited by HER2-Th (*p* < 0.001). Similarly, HER2-specific IgG antibody elicited by HER2-NitraTh was significantly higher than that elicited by HER2-Th group on days 21 and 28 (*p* < 0.01, Figure 4(b)). We sorted mice splenocytes on day 28 and enumerated HER2-specific human B cells by ELISpot assay. As shown in Figure 4(c), human IgM^+^ B cells against HER2 were significantly increased (*p* < 0.001) when DC-HIS mice were stimulated with HER2-NitraTh. HER2-NitraTh can significantly induce the generation of human IgG^+^ B cells because 36.3 ± 3.1 HER2-specific IgG-secreting B cells were present in every 2 × 10^5^ splenocyte (Figure 4(d)). The results above suggested that NitraTh-based vaccine can be used to induce autoantigen-specific IgG^+^ B cells in DC-HIS mice.

### 3.5. NitraTh Improved Activation of Peripheral Human T Cell and Induced Differentiation into Tfh Cells In Vivo

To understand how the NitraTh promoted the generation of IgG^+^ B cells further, we immunized the DC-HIS mice with HER2-NitraTh or HER2-Th and explored the effect of NitraTh on human T cell subsets. HER2-NitraTh and HER2-Th had no significant effect on the frequency of total human mononuclear cells (hCD45^+^) in spleen ([Supplementary-material supplementary-material-1]). The frequency of the CD4^+^ T cells in the HER2-NitraTh group was statistically higher than that in the control group (*p* < 0.05), whereas HER2-Th showed no effect on the proportion of human CD4^+^ T cells in the spleen (Figure 5(a)). The frequency of CD4^+^CD69^+^ T cells in HER2-NitraTh group was also significantly higher than that in HER2-Th group (*p* < 0.01, Figure 5(b)), thereby indicating that NitraTh improved the activation of CD4^+^ T cells. We also observed increased human T cell in the lymph nodes of HER2-NitraTh group (Figure 5(c)), thereby suggesting that the NitraTh may enhance the immune response in the peripheral immune organ.

HER2-NitraTh induced significantly higher percentage of human follicular helper T cells (Tfh) in the spleen of DC-HIS mice than HER2-Th (*p* < 0.01, Figure 5(d)). Tfh cells are critical in driving B cell proliferation, survival, and isotype class switching [17]. Consistent with this result, high B cell proportion was detected in the spleen of mice in the Her2-NitraTh group (Figure 5(e)). The immunohistochemical detection of the lymph node also revealed that additional B cells were present in the lymph nodes of HER2-NitraTh-immunized mice (Figure 5(f)). Our results indicated that human B cells can survive well and localize to peripheral lymphoid organs in DC-HIS mice under the support of Tfh cells induced by HER2-NitraTh.

### 3.6. B Cells Transferred into DC-HIS Mice Display Somatic Hypermutation and Undergo Antibody Class Switching in Stimulation with NitraTh-Based Vaccines

Given that HER2-NitraTh can induce HER2-specific IgG^+^ B cells in DC-HIS mice, we sorted CD3^−^CD19^+^CD27^+^HER2^+^ B cells from splenocytes of DC-HIS mice immunized with Her2-NitraTh ([Supplementary-material supplementary-material-1]). To characterize the correlation between somatic hypermutation rates of memory B cells and NitraTh further, we also sorted CD3^−^CD19^+^ naïve B cells from peripheral blood of the same donor for comparison ([Supplementary-material supplementary-material-1]). An identifiable PCR product was obtained from 9 CD3^−^CD19^+^ naïve B cells and 31 CD3^−^CD19^+^CD27^+^HER2^+^ B cells, respectively ([Supplementary-material supplementary-material-1]).

**Figure 6:**
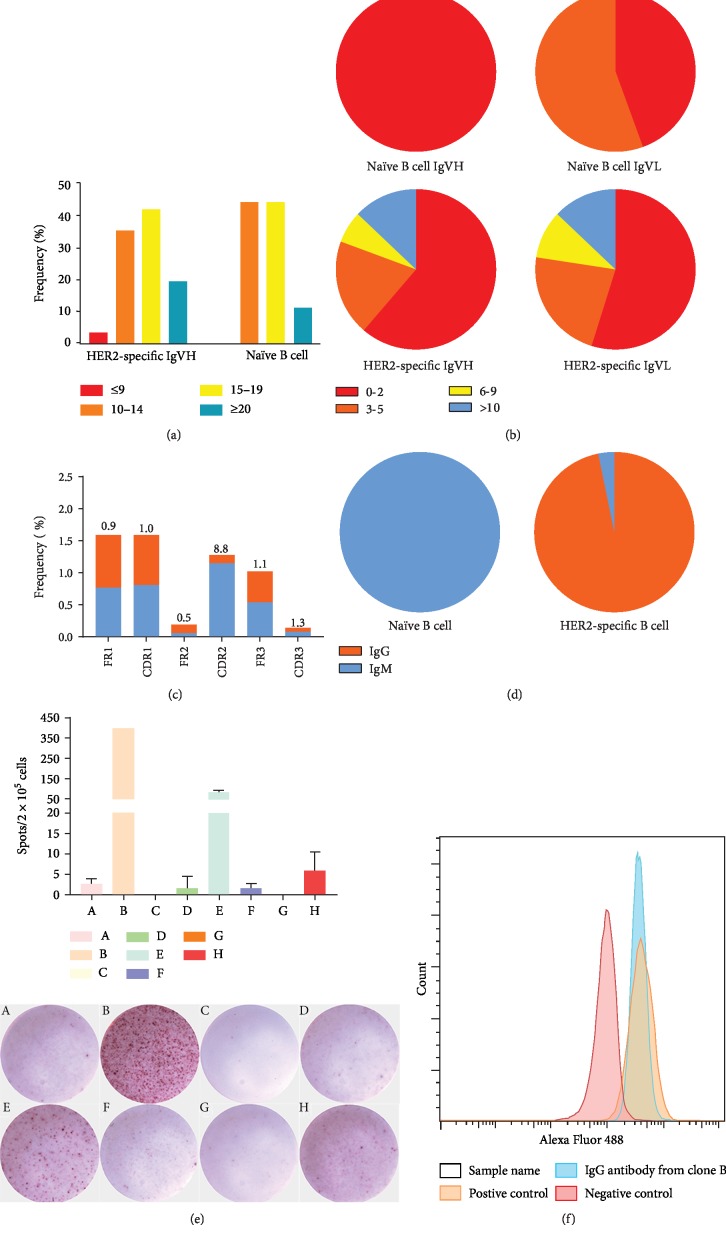
B cells transferred into HIS mice displayed somatic hypermutation and underwent antibody class switching in the stimulation of NitraTh-based vaccines. NCG mice were engrafted with PBMCs on day 0 and DCs on day 7 to build DC-HIS mice. Then, DC-HIS mice were immunized with HER2-NitraTh or HER2-Th on days 7 and 21. Splenocytes were harvested from vaccinated mice on day 28 for sorting CD3^−^CD19^+^CD27^+^HER2^+^ B cells. CD3^−^CD19^+^ naïve B cells were sorted from peripheral blood in the same donor. The VH and VL genes of these cells were amplified by RT-PCR with specific primers. (a) Bar graphs depicted the frequencies of the VH-CDR3 length with ≤9, 10 to 14, 15 to 19, and ≥20 amino acids (aas). (b) Pie charts showed the proportion of VH and VL with 0 to 1, 3 to 5, 6 to 9, and ≥10 somatic mutations. (c) The ratio of the replacement (blue bar) and silent mutations (orange bar) in IGHV-FWRs and CDRs was calculated in vaccinated mice as mutated nucleotides per total base pairs analyzed. The R/S ratio for each region was indicated. (d) Pie charts depicted the frequencies of the antibody gene types of naïve B cells and HER2^+^ B cells. (e) Eight IgG sequences from HER2^+^ B cells were cloned into pCDNA3.1 vector and transformed into the HEK293 cell line. Transfected HEK293 cells were tested by ELISpot assay to confirm that the antibodies encoded by the obtained sequences can bind with HER2. (f) FACS determined the specific binding capacity of the antibody produced by clone B to SK-BR-3.

Sequence analysis showed that although the difference in the average length of CDR3 between B cells from peripheral blood and HER2-NitraTh group (15.6 versus 16.3) was not significant, the latter had a broader range of the lengths of CDR3 than the former (Figure 6(a)). This result indicated that the B cell antibody library was increasingly diverse after the stimulation of HER2-NitraTh.

When comparing Ig variable region data to unmutated germ line forms, we observed that HER2-NitraTh group had a high level of hypermutation in the variable regions of VH and VL genes. The proportion of VH or VL with >10 mutations was ~13% (Figure 6(b)). We compared the ratio of replacement mutations to silent mutations (R/S) in CDRs and framework regions (FWRs). CDRs had a higher R/S ratio than FWRs (Figure 6(c)), thereby indicating that antigen-driven selection occurred in DC-HIS mice immunized with HER2-NitraTh. The antibody genes obtained from peripheral blood were all IgM class, while the antibody genes obtained from HER2-NitraTh-immunized mice were almost all IgG class (Figure 6(d)). This result indicated that human B cells underwent antibody class switching to produce specific IgG antibodies in DC-HIS mice.

To confirm that the antibody encoded by the obtained sequences can bind to HER2, we cloned 8 paired VH and VL genes amplified from single B cells and expressed antibodies in the HEK293 cell line. The results of ELISpot assay shown that six out of the eight clones produced antibodies that bind to the B epitope of HER2. Among these clones, clone B produced the highest number of spots, thereby suggesting that clone B would have a higher expression level than the other clones (Figure 6(e)). Therefore, we purified the antibody produced by clone B and tested whether the antibody can bind to the extracellular region of HER2 on the cell membrane by FACS. As shown in Figure 6(f), IgG antibody from clone B can bind to SK-BR-3 cells with high HER2 expression, and the difference was not significant compared with the positive control.

## 4. Discussion

In this study, we established an efficient strategy on the basis of the combination of single B cell antibody technology and NitraTh-based vaccine to obtain fully human antibody gene rapidly. NitraTh can help self-antigens to break immune tolerance. The immunization of DC-HIS mice with HER2-NitraTh allowed us to successfully isolate HER2-specific B cells that underwent somatic mutation and class switching. We obtained 30 fully human IgG antibody sequences from sorted B cells and confirmed that antibodies that are coded by these sequences can bind to HER2.

The single B cell technology is limited by the difficulty of obtaining single B cells against autoantigens. Hence, breaking self-tolerance is a challenge of applying single-B cell technology to the development of fully human antibody drugs. The introduction of non-self-components, such as unnatural aas, into self-proteins is a common strategy to address this challenge. The *p*-nitrophenylalanine mutant of the self-proteins, TNF-*α*, epidermal growth factor, retinol-binding protein, and complement factor 5a induce specific antibody responses to their respective endogenous counterparts [11–13, 18]. Meanwhile, the loss of immunological tolerance induced by *p*-nitrophenylalanine was position- and MHC class II-dependent. The substitution of *p*-nitrophenylalanine for Lys^11^, instead of Gln^21^, in TNF-*α* breaks immunological tolerance against the self-protein [11]. As a result, the appropriate substitution sites of *p*-nitrophenylalanine should be screened for each self-protein, which is generally time-consuming and labor-intensive.

To address this problem, we found a simpler and more effective way to overcome immune tolerance and stimulate the generation of autoantigen-specific B cells than directly incorporating *p*-nitrophenylalanine into autologous proteins. The universal Th epitope is a sequence of 13 aas that can bind to different mouse MHC II and human HLA II molecules, promote proliferation and differentiation of CD4^+^ T cells, and help B epitopes to produce high-titer antibodies [19, 20]. Changing any amino acid in the core sites of this universal Th epitope can significantly affect the affinity of the Th epitope for MHC II or T cell receptor [21].

In our previous work, NitraTh, a non-self T cell epitope formed by substituting *p*-nitrophenylalanine for amino acid 11 at the universal T cell epitope can significantly enhance the immunogenicity of HER2 vaccine in mice with different MHC II backgrounds and induce strong specific B cell responses [14]. Therefore, the fusion of the NitraTh and autoantigens may be a more convenient method of breaking self-tolerance.

In the present work, we further confirmed that NitraTh can assist in breaking immune tolerance in the context of human immune system. Considering the differences of MHC class II molecules between human and murine, we first selected the most suitable residue for *p*-nitrophenylalanine incorporation. The newly screened NitraTh, which replaced amino acid 5 of the universal T cell epitope with *p*-nitrophenylalanine, has a significantly enhanced capacity of activating human naïve CD4^+^ T cells (Figure 1(b)). HER2-NitraTh induced a significantly higher level of human HER2-specific IgM *in vitro* than HER2-Th, which contained universal T helper epitope without *p*-nitrophenylalanine (Figures 2(a) and 2(b)). Then, we tested this approach in the context of a second protein (CB1) and found that CB1-NitraTh can remarkably induce human CBR1-specific IgM^+^ B cell responses *in vitro* (Figures 2(c) and 2(d)). This result indicated that this methodology can be generalized to different self-proteins. NitraTh also retained extremely broad pattern of HLA II molecular binding, which was extremely important in establishing a strategy applicable to different individuals. A total of >85% of samples with different HLA-DR haplotypes had a strong proliferative response after stimulation with NitraTh-based vaccines (Table 1). The genotypes of response included HLA-DR9 alleles, which are among the most prevalent DR alleles in the Chinese population (28.3%) [22].

Although NitraTh-based vaccines can break self-tolerance and induce IgM-secreting B cells *in vitro*, directly inducing IgG-secreting B cells *in vitro* was extremely difficult mainly because of the lack of a microenvironment that supports B cell differentiation and maturation [23]. To provide a stable microenvironment that supports the maturation of IgG-secreting B cells, we constructed a novel HIS mice model on the basis of DC cotransplantation.

The HIS mice model is a humanized mouse model produced by reconstituting a functional human immune system in immunodeficient mice and has been widely used in vaccines, tumors, and infectious diseases [24, 25]. The human immune system reconstructed in immunodeficient mice can be stimulated by antigens to produce specific immune responses [26–28]. However, a gap of immune system between human and HIS mice that directly transplanted PBMCs was still present. The B cell response in HIS mice is not robust, and the scarcity of IgG-secreting B cells makes them extremely difficult to be identified and isolated [29]. Consistent with these findings, our results also showed that the KLH-specific IgG level in the sera of HIS mice immunized with KLH was not significant (Figure 3(c)).

The poor IgG^+^ B cell response may be because mouse DC-activated human T helper cells do not support human B cells [30]. Human antigen-presenting cells are extremely rare in HIS mice engrafted with PBMCs [15, 16]. Thus, to improve the generation of antigen-specific IgG^+^ B cells, we cotransplanted antigen-loaded autologous DC in the HIS mice. The mice immunized with KLH-loaded DCs, instead of KLH alone, displayed significantly high serum IgG titers reacting against KLH (Figure 4(b)). Similar results can also be reproduced on another model antigen, such as OVA (Figure 4(d)). Hence, the novel HIS mice model on the basis of DC cotransplantation provided a reliable approach to acquire human IgG^+^ B cells. To obtain human IgG^+^ B cells against self-protein HER2, we immunized DC-HIS mice with HER2-NitraTh vaccine. The results showed that DC-HIS mice immunized with HER2-NitraTh displayed significantly high serum IgG titers (Figure 4(b)). Specific human IgG^+^ B cells were detected in the spleen (Figure 4(d)) of immunized mice, which reached our expectations.

Our study provided additional insights into the impact of NitraTh-based vaccine on human lymphocytes *in vivo*. We discovered that NitraTh can promote the proliferation and activation of human CD4^+^ T cells in mice (Figures 5(a)–5(b)). The differentiation of the Tfh cells also occurred in mice after stimulation with NitraTh-based vaccines (Figure 5(d)). Tfh cells provided help to B cells through costimulatory molecules and cytokines [31], which may lead the survival of B cells in the spleen and lymph nodes 1-month postengraftment (Figures 5(e) and 5(f)). This phenomenon is important for efficient B cell activation and selection.

Finally, we isolated approximately 16 CD3^−^CD19^+^CD27^+^HER2^+^ B cells per 1 × 10^5^ splenocytes and successfully acquired 31 antibody variable region sequences. Sequence analysis displayed the sorted HER2-specific B cells had accumulated more VH gene and VL gene mutations than naïve B cells (Figure 6(b)). We also observed a high R/S radio in CDRs than in FWRs. Typically, a high R/S ratio in the CDRs of variable region genes is the molecular sign of antigenic selection and affinity maturation. The basic assumption of this statistical analysis is that antigenic selection favors replacement mutations in CDRs and silent mutations in FWRs [32].

Class switching is a sign of robust B cell response and occurs after the activation of a mature B cell via its membrane-bound antibody molecule to generate the different classes of antibody [33]. According to the NitraTh-based vaccines and the construction of the DC-HIS mice model, the antibodies we obtained almost all belonged to IgG class (30/31), thereby suggesting that the humoral immune response we induced in DC-HIS mice was extremely similar to the natural immune response in humans. Moreover, our method requires only 6-8 weeks to obtain human antibody genes specific to the target protein, which is more time-saving and convenient than the transgenic mouse or phage display platform [1, 34, 35].

In summary, we attempted to combine NitraTh-based vaccines with DC-HIS mice model to build a common platform that can acquire IgG^+^ B cells for the production of fully human antibodies against self-protein.

## 5. Conclusions

NitraTh-based vaccines can induce autoantigen-specific antibodies. We also developed a DC-HIS mice model that can support humoral immune responses to various antigens and produce antigen-specific IgG^+^ B cells. In summary, we provided a convenient and effective method in acquiring fully human antibody genes against self-proteins, which can be used to develop therapeutic antibody drugs.

## Figures and Tables

**Table 1:** HER2-NitraTh can activate CD4^+^ T cells with different HLA class II haplotypes.

Sample	Genotype		Proliferation rate of naïve CD4^+^ T cells (%)	
	HLA-DR	HLA-DQ	Blank	HER2-NitraTh
1	DRB1^∗^01 DRB1^∗^09	DQB1^∗^03 DQB1^∗^05	1.69 ± 0.24%	18.77 ± 2.28% (^∗∗^)
2	DRB1^∗^11 DRB1^∗^15	DQB1^∗^03 DQB1^∗^06	2.69 ± 0.73%	14.67 ± 1.72% (^∗∗^)
3	DRB1^∗^07 DRB1^∗^14	DQB1^∗^02 DQB1^∗^05	1.64 ± 0.67%	18.6 ± 5.78% (^∗^)
4	DRB1^∗^08 DRB1^∗^15	DQB1^∗^03 DQB1^∗^06	4.40 ± 0.84%	15.30 ± 1.57% (^∗∗^)
5	DRB1^∗^07 DRB1^∗^15	DQB1^∗^02 DQB1^∗^06	2.09 ± 0.84%	7.34 ± 1.47% (^∗^)
6	DRB1^∗^04 DRB1^∗^09	DQB1^∗^03 DQB1^∗^03	3.04 ± 1.67%	11.13 ± 2.38% (n.s.)
7	DRB1^∗^01 DRB1^∗^12	DQB1^∗^03 DQB1^∗^05	1.62 ± 0.26%	16.06 ± 2.72% (^∗^)

Naïve CD4^+^ T cells were sorted from peripheral blood and stimulated with HER2-NitraTh for 7 days, and the proliferation rate was measured by FACS. Meanwhile, the HLA-DR and HLA-DQ loci of donor PBMCs were detected by PCR amplification with sequence-specific primers.

## Data Availability

The data used to support the findings of this study are available from the corresponding author upon request.

## Abbreviations

HER2: Human epidermal growth factor receptor 2
CB1: Cannabinoid receptor 1
HIS: Human immune system mice
mAbs: Monoclonal antibodies
TNF-*α*: Tumor necrosis factor alpha
PBMCs: Peripheral blood mononuclear cells
DCs: Dendritic cells
KLH: Keyhole limpet hemocyanin
OVA: Chicken ovalbumin
Tfh: Follicular helper T cells
CDR: Complementarity-determining region
VH: Heavy-chain variable regions
VL: Light-chain variable regions
R/S: Replacement mutations to silent mutations
FWR: Framework regions.

## Supplementary Materials

Supplementary Materials Figure S1: the percentage of human total mononuclear cells (gated as hCD45+) in the spleen of the DC-HIS mice that were immunized with HER2-NitraTh or HER2-Th. Figures 2: single-cell sorted HER2-specific B cell from DC-HIS mice that were immunized with HER2-NitraTh to obtain antibody sequences.
