# Supplementary material for: Efficient Acquisition of Fully Human Antibody Genes against Self-Proteins by Sorting Single B Cells Stimulated with Vaccines Based on Nitrated T Helper Cell Epitopes
Source: J Immunol Res. 2019 Dec 30;2019:7914326. doi: 10.1155/2019/7914326 (PMC7012236; doi:10.1155/2019/7914326)
Supplement: Supplementary Materials — Figure S1: the percentage of human total mononuclear cells (gated as hCD45+) in the spleen of the DC-HIS mice that were immunized with HER2-NitraTh or HER2-Th. Figures 2: single-cell sorted HER2-specific B cell from DC-HIS mice that were immunized with HER2-NitraTh to obtain antibody sequences. [file 7914326.f1.docx]

**
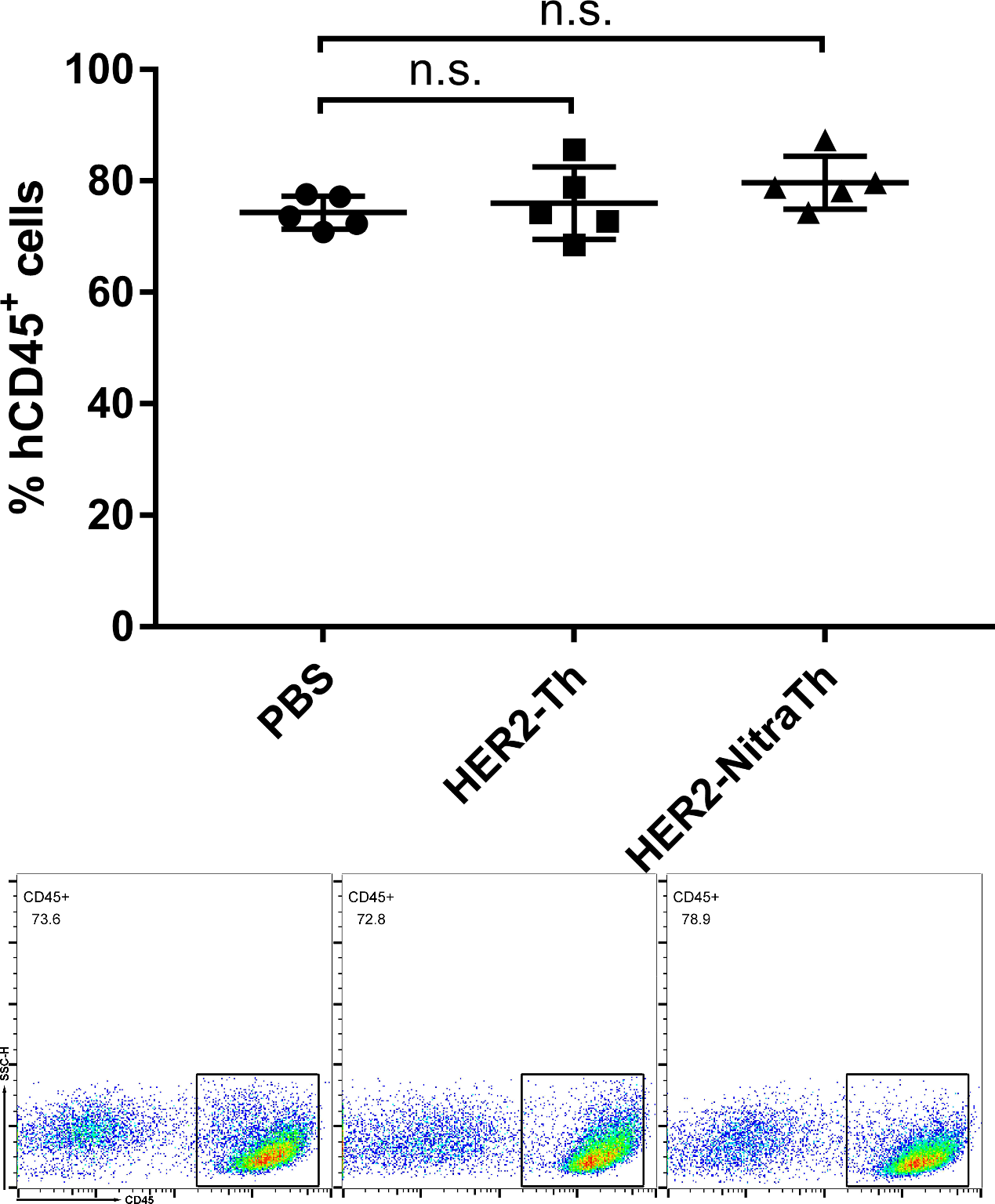
**

**Supplement Fig. 1.** NCG mice were engrafted with PBMCs on day 0 and DCs on day 7 to build DC-HIS mice. Then DC-HIS mice were immunized with HER2-NitraTh or HER2-Th on day 7 and 21. Splenocytes were harvested from vaccinated mice on day 28. Data are expressed as mean ± SD (n = 5). FACS determined the percentage of human total mononuclear cells (gated as hCD45^+^) in the spleen.

**
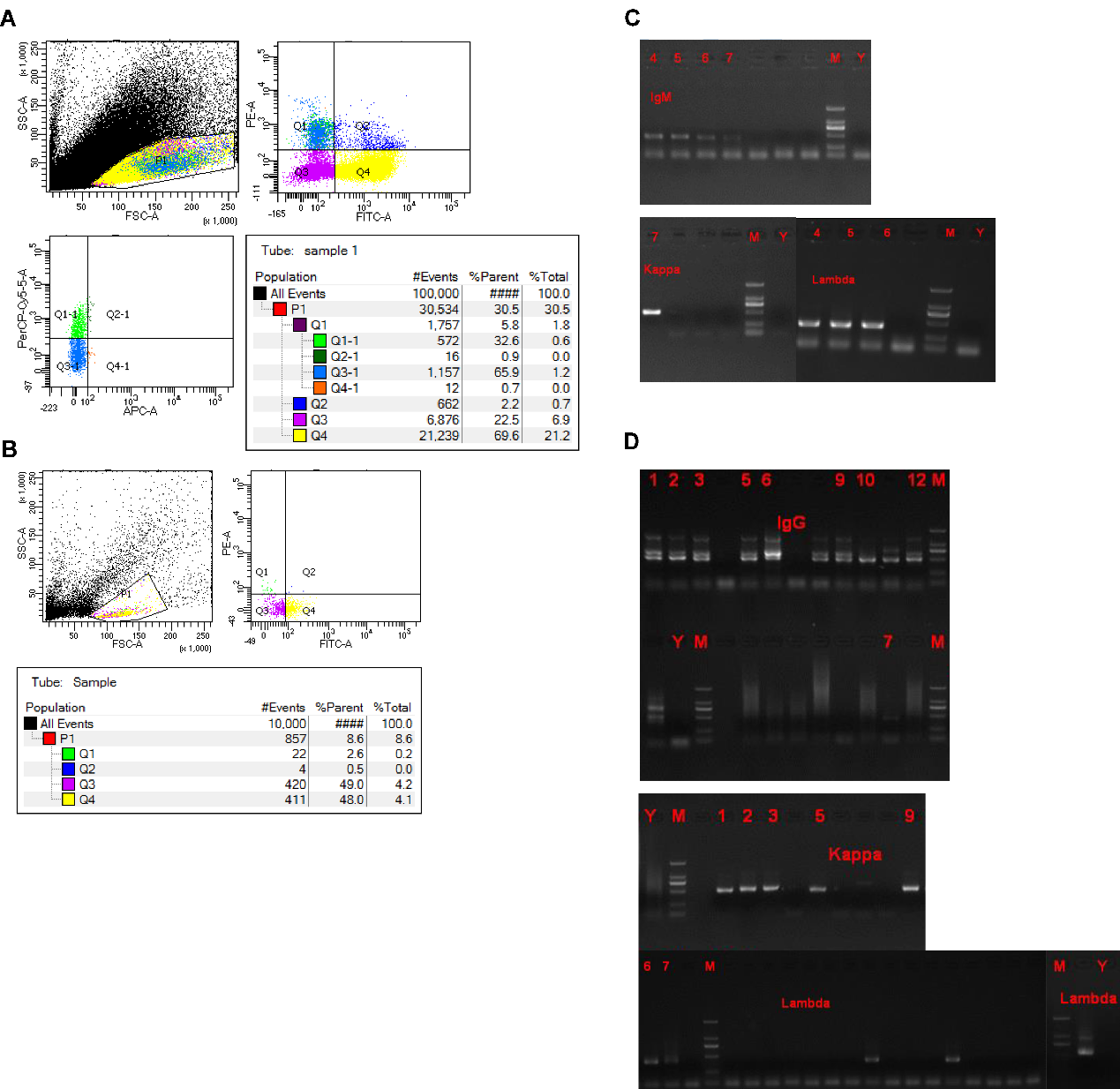
**

**Supplement Fig. 2.** **Single-cell sorted naïve B cells and HER2-specific B cell to obtain antibody sequences.** (A) CD3^-^CD19^+^CD27^+^HER2^+^ B cells were sorted from the splenocytes of DC-HIS mice that immunized with HER2-NitraTh. (B) CD3^-^CD19^+^ naïve B cells were sorted from peripheral blood in the same donor. Sequences of VH and VL were obtained by performing RT-PCR with a cocktail of appropriate family-specific primer. Representative experiments were shown. (C) Electropherograms of PCR amplification products from naïve B cells. (D) Electropherograms of PCR amplification products from HER2-specific B cells. M: D2000maker, Y: Negative control. We only mark samples that have been successfully sequenced.
